# Diagnostic sensitivity of formalin-fixed faecal microscopy for the detection of soil-transmitted helminths

**DOI:** 10.1093/trstmh/traf011

**Published:** 2025-02-08

**Authors:** Andrew Larkins, Boualay Keokhamphavanh, Breanna Knight, Kelly Taggart, Sarah Keatley, Bounnaloth Insisiengmay, Amanda Ash

**Affiliations:** School of Medical, Molecular and Forensic Sciences, Murdoch University, 90 South St, Perth 6150, Australia; Centre for Biosecurity and One Health, Harry Butler Institute, Murdoch University, 90 South St, Perth 6150, Australia; Department of Communicable Disease Control, Ministry of Health, Rue Simeuang, Vientiane 01030, Lao PDR; School of Medical, Molecular and Forensic Sciences, Murdoch University, 90 South St, Perth 6150, Australia; Centre for Biosecurity and One Health, Harry Butler Institute, Murdoch University, 90 South St, Perth 6150, Australia; School of Medical, Molecular and Forensic Sciences, Murdoch University, 90 South St, Perth 6150, Australia; Centre for Biosecurity and One Health, Harry Butler Institute, Murdoch University, 90 South St, Perth 6150, Australia; School of Medical, Molecular and Forensic Sciences, Murdoch University, 90 South St, Perth 6150, Australia; Centre for Biosecurity and One Health, Harry Butler Institute, Murdoch University, 90 South St, Perth 6150, Australia; Department of Communicable Disease Control, Ministry of Health, Rue Simeuang, Vientiane 01030, Lao PDR; School of Medical, Molecular and Forensic Sciences, Murdoch University, 90 South St, Perth 6150, Australia; Centre for Biosecurity and One Health, Harry Butler Institute, Murdoch University, 90 South St, Perth 6150, Australia

**Keywords:** Bayesian analysis, diagnostic tests, latent class analysis, microscopy, neglected tropical diseases, parasitology

## Abstract

**Background:**

Faecal microscopy is the mainstay of soil-transmitted helminth diagnosis and commonly completed on formalin-fixed samples when resources are insufficient to analyse fresh samples. This study assessed the diagnostic sensitivity of microscopic techniques using formalin-fixed samples.

**Methods:**

Formalin-fixed faecal samples from 574 individuals were tested by the formalin-ethyl acetate concentration technique (FECT), Malachite smear, McMaster and McMaster2 methods. Agreement between tests was assessed by Kappa. Bayesian latent class models and a composite reference standard estimated the diagnostic sensitivity of each test.

**Results:**

Moderate-to-good agreement between tests was observed for *A. lumbricoides*. Agreement was poorer for hookworm and *Trichuris trichiura*. The FECT (72.70%, credible interval [CrI]: 68.92–76.56%) and McMaster2 method (67.93%, 95% CrIs: 62.41–73.31%) had the highest sensitivities for *A. lumbricoides*. For hookworm, the McMaster2 method (70.56%, 95% CrIs: 64.10–76.96%) was more sensitive than all other tests. For *T. trichiura*, the McMaster (90.10%, 95% CrIs: 83.29–94.67%) and McMaster2 (89.3%, 95% CrIs: 82.28–94.52%) methods were the most sensitive.

**Conclusions:**

The McMaster2 method is a viable alternative to FECT and provides important information on the intensity of infection. The effect of formalin-fixation on test performance may not be as great as previously assumed. This study reports formalin-fixed sensitivities similar to previous estimates using fresh samples.

## Introduction

Soil-transmitted helminths (STHs) are a group of intestinal parasites in which contaminated soil plays an important role in their lifecycles. The main STHs are *A. lumbricoides*, hookworm (*Ancylostoma duodenale* and *Necator americanus*) and *Trichuris trichiura*. There are other helminths that may be included in this group, such as *Strongyloides stercoralis*; however, this paper focuses on the three traditional STHs. Soil-transmitted helminths continue to place a substantial burden on endemic communities that are regularly found in low- and middle-income countries. Infections may lead to anaemia, malnutrition, impaired physical and cognitive development, abdominal pain and diarrhoea.^1^ Preventive chemotherapy or mass drug administration is the cornerstone of control programmes and is often supported by commercial pharmaceutical donations.^2^ These programmes, in conjunction with wide-sweeping improvements to water, sanitation and hygiene infrastructure and economic development, have successfully led to improved health in many communities across the globe.^3^ Despite the progress made there is still much work to be done and the Global Burden of Disease programme estimated that close to 1 billion people were still infected with STHs in 2021.^4^

Diagnostics are critical to control programmes and the World Health Organization (WHO) recommends preventative chemotherapy in at-risk groups when the prevalence of STHs is >20%.^5^ Faecal microscopy is the mainstay of STH diagnosis due to its low cost and accessibility,^6^ and procedures using fresh faecal samples have been recommended by the WHO since the 1990s, with the Kato-Katz method being the most common.^7,8^ However, faecal microscopy of fresh samples must be completed rapidly for accurate results. For example, it is recommended that the Kato-Katz method is completed within 30–60 min for hookworm and 24 h for schistosomiasis.^8^ These recommendations present a substantial logistical challenge in the rural and remote communities that are often most affected by STHs. In large surveys, it means that a large number of personnel are required to analyse the samples in the field, as transporting the samples to laboratory would take longer than recommended and require temperature control to preserve the samples. As such, some countries have chosen to use methods that can be applied to formalin-fixed samples and do not require rapid field analysis during large-scale surveys.^9^

Formalin-fixed samples are often preferred in large surveys as they can be stored and tested at a later date and over a longer period at a central laboratory. Global meta-analysis suggested that a formalin-fixed diagnostic method, the formalin-ethyl acetate concentration technique (FECT), may have similar performance to the fresh Kato-Katz depending on the setting.^10^ The McMaster method is another microscopic test that can be completed on formalin-fixed samples. It is less commonly performed in medical laboratories; however, it has been applied extensively in the veterinary sector and provides the added benefit of calculating standardised intensity of infection in the form of eggs per gram (EPG).^8,11^ The McMaster method has been included in the latest WHO bench aids for the diagnosis of intestinal parasites and has been applied previously during large STH surveys.^8,10–14^ Previous studies assessing the performance of the McMaster method have generally been limited to its use on fresh samples. Only one published STH study assessing the McMaster method on formalin-fixed samples is known to the authors.^11^ Due to the lack of sensitivity estimates for the McMaster method when using formalin-fixed samples, the aim of this study was to compare the McMaster method and FECT using formalin-fixed samples during a large-scale survey.

## Materials and methods

### Data collection

This diagnostic study was embedded prospectively within a research project investigating risk mapping for *Taenia solium* in the Lao People's Democratic Republic (Lao PDR). The research project purposively selected 42 study villages from three districts in northern Laos: Viengkham district, Luang Prabang province; Mai district, Phongsaly province; and Nga district, Oudomxay province. Villages were selected based on a semiquantitative risk assessment for *T. solium* conducted by provincial health staff. Sample sizes were calculated for the estimation of *T. solium* prevalence within villages and the comparison of high- and low-risk villages. Participant enrolment and convenient sample collection occurred in villages from May 2022 to April 2023. Information sessions were held in each village and individuals had the opportunity to return faecal samples 12–24 h after each session. Some villages were visited more than once due to their location, providing some individuals with an additional opportunity to return samples. Any individual residing in the village at the time of the information session and attending or hearing about the information session was considered eligible to participate. Approximately 2 g of faecal material was taken from returned samples and was preserved in 10% formalin (two parts faeces:eight parts formalin), macerated with a wooden spatula and shaken to encourage homogenisation. A random 20% of samples from six villages in one district were duplicated for testing by different microscopy methods at separate laboratories and these villages are considered the population of this study.

### Laboratory testing

All samples were analysed by FECT at the Lao Ministry of Health within 2–4 mo of collection. The random duplicates were tested by the McMaster method without straining, novel McMaster (McMaster2) method and Malachite green smear at Murdoch University 3–12 mo after collection.^8,15^

The FECT and McMaster method were completed as previously published and chosen for their ability to be performed on formalin-fixed samples.^8^ The novel McMaster2 protocol was developed due to the larger project's aim of detecting *T. solium* and was conducted by counting both the top and bottom focal layers of the McMaster grid. In the standard McMaster, only the top focal layer is counted, where grid lines then air bubbles are the point of focus. The decision to count both the top and bottom layers was made as the authors’ experience suggested that *Taenia* eggs often remain at the bottom layer of the McMaster grid if read too quickly and may be missed using the standard method. This alteration was expected to increase the sensitivity of the McMaster method for *Taenia* spp.^16^ The Malachite smear was completed to detect protozoans that may be missed by other methods. However, it is methodologically analogous to the direct smear that is used during STH surveys, with the addition of a Malachite green stain. The Malachite smear was completed as published.^15^

### Diagnostic agreement

Test data were summarised by result into contingency tables and apparent prevalence was estimated for each test with 95% CrIs estimated by Jeffrey's method.^17^ A composite reference standard (CRS) was created by considering the tests in parallel. Individuals that tested positive to any test were considered positive by the CRS and an individual had to test negative to all tests to be considered negative. Agreement between tests was assessed using Kappa and calculated in R (version 4.2.3, R Statistical Foundation, Vienna, Austria) by the epi.kappa function of the EpiR package.^18^ Kappa is a measure of the agreement between tests beyond random chance and is commonly interpreted with the following thresholds: K<0.20 poor; 0.21–0.40 fair; 0.41–0.60 moderate; 0.61–0.80 good; and 0.81–1.00 very good agreement.

Intensity of infection was recorded as EPG by the McMaster and McMaster2 methods. Intensity category was assigned using WHO intensity-level thresholds.^19^ Agreement between EPGs was assessed using Kendall's tau. The arithmetic mean EPG was calculated and compared using a one-sided Wilcoxon signed rank test. Because the McMaster2 method incorporates the standard McMaster count, the alternative hypothesis evaluated was that the mean McMaster2 EPG was greater than the mean McMaster EPG as it is not possible for the McMaster2 intensity to be less than the McMaster intensity. The FECT and Malachite smear are unable to provide quantitative estimates of EPG and could not be included in this comparison. The presence of eggs on the top and bottom layers of McMaster slides was assessed by contingency table to examine how many additional cases were detected by examining the bottom layer during the McMaster2 method. The difference in apparent prevalence between McMaster and McMaster2 methods was assessed for significance using a one-sided Z-test where the alternative hypothesis was that the McMaster2 apparent prevalence was greater than the McMaster apparent prevalence.

### Diagnostic sensitivity

Diagnostic sensitivity is the proportion of diseased individuals that are correctly classified as positive by a diagnostic test.^20^ When there is no gold standard to correctly determine the true disease status of an individual, CRS or Bayesian latent class models (BLCMs) may be used.^21^ In the first instance, the sensitivity of each test was compared with the CRS with 95% CrIs estimated using Jeffrey's method.^17^ In the second instance, BLCMs were developed for each STH and their posterior distributions reported by the median and 95% credible interval (CrI) (Online Appendix 1).

Bayesian latent class models assume that the standard contingency tables that compare diagnostic tests follow a multinomial distribution based on prevalence and test performance.^22^ Covariance terms can be included when there is conditional dependency between tests and this allows for factors other than true infection status to influence both test outcomes simultaneously.^23^ In this study, all methods are based on the observation of STH eggs by faecal microscopy and testing should be considered conditionally dependent. As such, pairwise covariance terms were included in all BLCMs (Online Appendix 1). A BLCM assessing three tests with conditional pairwise dependence has seven degrees of freedom, 13 parameters and requires information on at least six parameters to ensure it is identifiable.^24^ In the case of faecal microscopy, the methods have been shown to be extremely specific and sensitivity is the practical metric of interest.^10,25^ By fixing the specificity and the related covariance parameters in a BLCM, the degrees of freedom remain at seven and the number of parameters is reduced to seven, making the model identifiable without the need for informed prior distributions. Specificities for all tests were fixed at 99.60% for *A. lumbricoides*, 98.00% for hookworm and 97.50% for *T. trichiura* based on published global meta-analysis.^10^

Informative prior distributions for test sensitivity were based on the median and lower 95% CrIs from global meta-analysis of populations with low-intensity of infection^10^ (Table 1). The betabuster function from the EpiR package was used to convert these values into beta distributions for use in the BCLMs.^18^ Values for FECT were taken as published, while the published McMaster values were altered as the published values reflected analysis of fresh faecal samples. The sensitivity of fresh faecal microscopy is assumed to be higher than that of formalin-fixed and the published lower CrI was decreased by 10 percentage points to widen the beta distribution and increase prior uncertainty around the sensitivity of the McMaster and McMaster2 methods. The sensitivity of the Malachite smear has not been formally assessed for STHs; however, it is methodologically equivalent to a direct smear and was assumed to perform comparably with the direct smear assessed by Nikolay, Brooker,^10^ with the exception of formalin fixation. The published lower CrI was again decreased by 10 percentage points to reflect the uncertainty around the change in sensitivity due to formalin-fixation. Informative prior distributions for each STH prevalence were taken from the latest national helminth survey in Lao PDR.^9^ The published lower CI was decreased by 10 percentage points to increase uncertainty as the national survey included few villages from this study's population. If any publisher lower CI was <10% then the minimum input for the betabuster function was set at 0.001%.

**Table 1.** Median and 95% CrIs for informed prior beta distributions based on Nikolay, Brooker.^9^ Beta(1,1) distributions were used for all parameters in the vague BLCM

Parameter	*A. lumbricoides*	Hookworm	*T. trichiura*
FECT sensitivity	51.10% (21.40–80.25%)	38.95% (33.50–44.59%)	22.72% (10.60–39.11%)
Malachite sensitivity	50.00% (2.50–97.50%)	50.00% (2.50–97.50%)	62.59% (51.90–72.52%)
McMaster sensitivity	48.98% (27.20–71.04%)	35.45% (17.90–56.23%)	74.65% (60.00–86.26%)
McMaster2 sensitivity	48.98% (27.20–71.04%)	35.45% (17.90–56.23%)	74.65% (60.00–86.26%)
Prevalence	15.95% (0.78–58.08%)	22.74% (10.90–38.62%)	10.30% (0.50–41.62%)

BLCM: Bayesian latent class model; CrI: credible interval; FECT: formalin-ethyl acetate concentration technique.

A vague BLCM was analysed to assess the impact of the informed prior distributions from the literature. In this model, beta(1,1) distributions were used for all test sensitivities and prevalence. The beta(1,1) is a flat or uniform probability distribution ranging from zero to one. Vague priors have minimal impact on the posterior distribution and allow for the provided data to have a larger impact on inference. The same initial values were set for all models and models were run for varying length with 10% burn-in until convergence was confirmed by visual examination of trace plots, a Brooks–Gelman–Rubin diagnostic <1.05 and effective sample sizes >4000 for all model parameters.^26^ All BLCM analyses were conducted in RStudio using Just Another Gibbs Sampler.^27^

## Results

### Participants and test results

**Figure 1.**
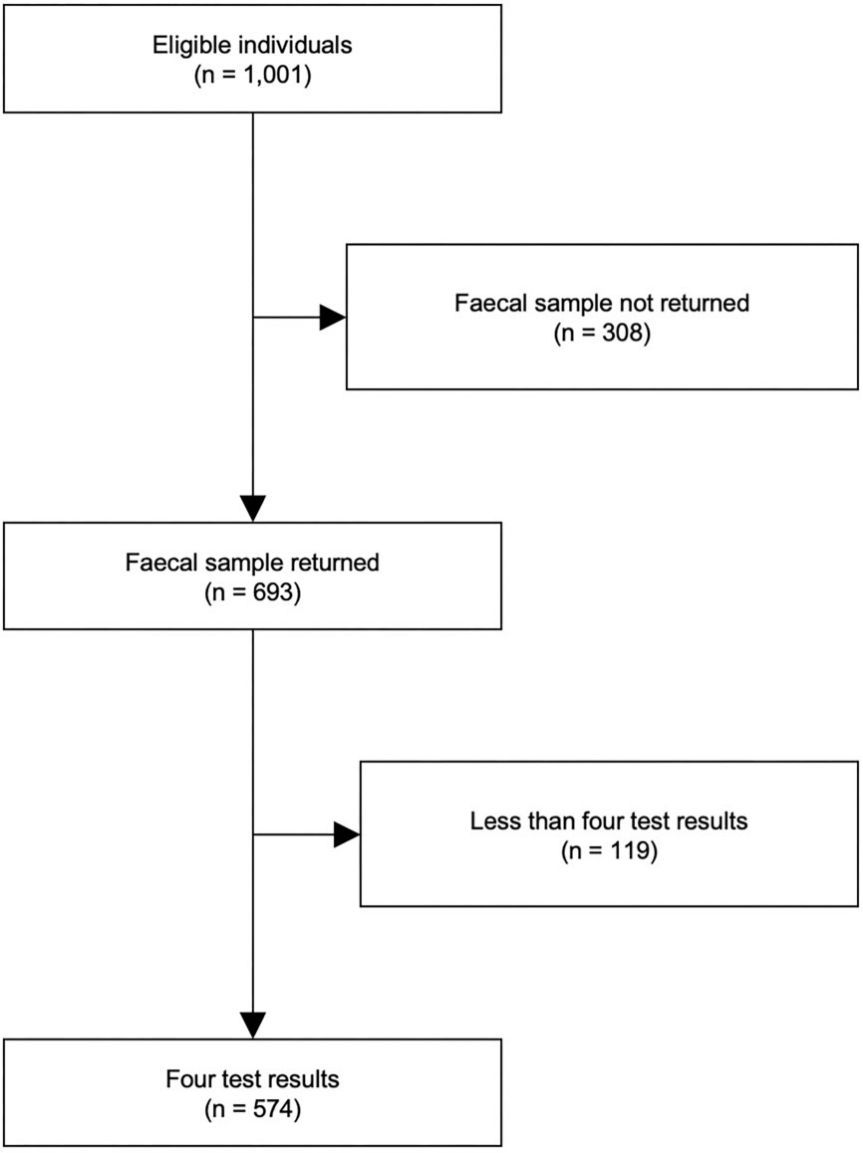
Participant flowchart.

A total of 1001 individuals attended information sessions in the six participating villages with 693 (69.23%) returning faecal samples. Of the returned samples, 574 (82.83%) had the FECT, Malachite smear, McMaster and McMaster2 methods performed (Figure 1). More than one-half (56.10%) of participants were female and the median age was 30 (range: 6–80) y. Test positivity varied with 48.61, 67.94 and 13.41% of individuals testing positive to ≥1 tests for *A. lumbricoides*, hookworm and *T. trichiura*, respectively (Online Appendix 2). However, there were sufficient data across all test results and STH combinations to proceed with BLCMs. Stratification by McMaster and McMaster2 intensity showed that almost all individuals had low-intensity infections and there were insufficient data to stratify BLCMs by intensity category^19^ (Table 2).

**Table 2.** Summary of McMaster2 intensity by WHO intensity-level threshold

Method	Intensity category^1^	*A. lumbricoides*	Hookworm	*T. trichiura*
McMaster	No infection	412 (71.78%)	362 (63.07%)	502 (87.46%)
	Light	153 (26.66%)	212 (36.93%)	72 (12.54%)
	Moderate	9 (1.57%)	0 (0.00%)	0 (0.00%)
	Heavy	0 (0.00%)	0 (0.00%)	0 (0.00%)
McMaster2	No infection	360 (62.72%)	302 (52.61%)	502 (87.46%)
	Light	205 (35.71%)	272 (47.39%)	72 (12.54%)
	Moderate	9 (1.57%)	0 (0.00%)	0 (0.00%)
	Heavy	0 (0.00%)	0 (0.00%)	0 (0.00%)
Total		574 (100.00%)	574 (100.00%)	574 (100.00%)

EPG: eggs per gram.

1 *A. lumbricoides*: light: 1–4999 EPG, moderate: 5000–49 999 EPG, heavy: ≥50 000 EPG. Hookworm: light: 1–1999 EPG, moderate: 2000–3999 EPG, heavy: ≥4000 EPG. *T. trichiura*: light: 1–999 EPG, moderate: 1000–9999 EPG, heavy: ≥10 000 EPG.^18^

### Diagnostic agreement

**Figure 2.**
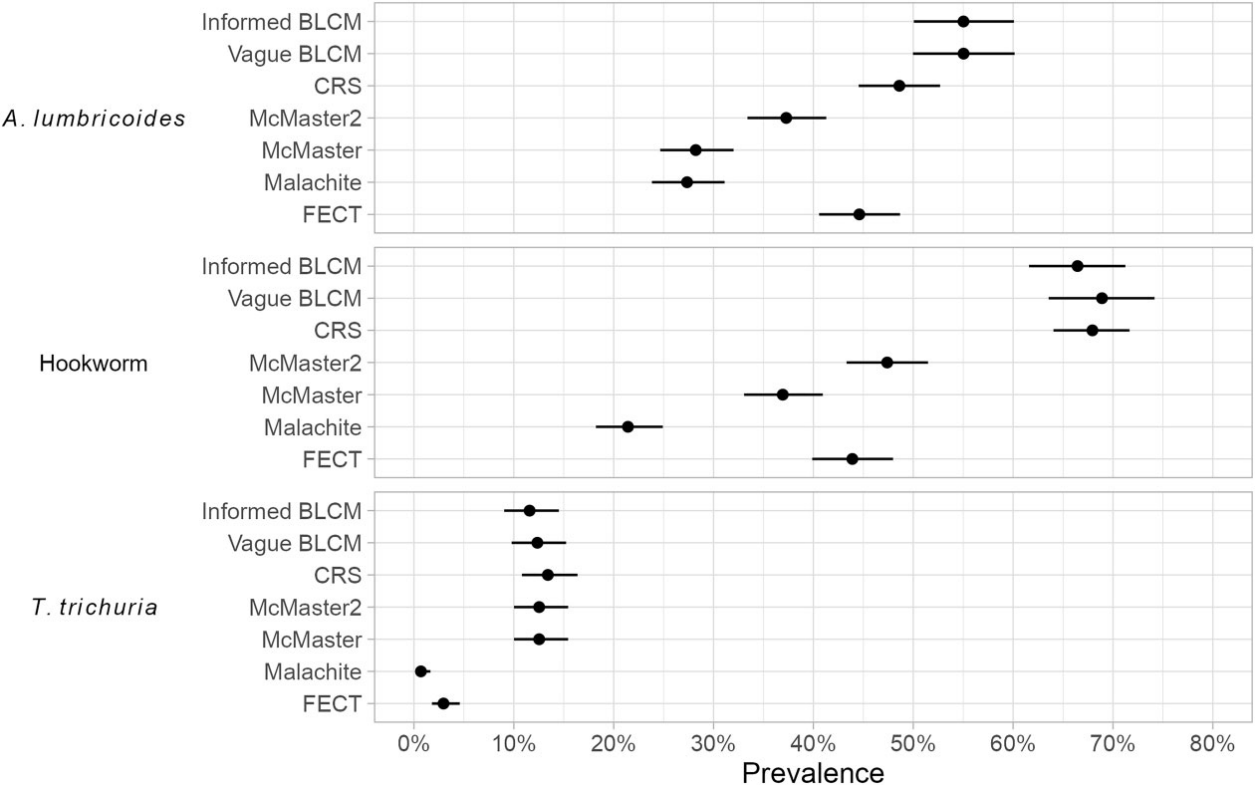
Prevalence of *A*. lumbricoides, hookworm and *T. trichuria* in the study population. Prevalence from Bayesian latent class models (BLCMs) is the estimated true prevalence whereas prevalence from all other methods is the estimated apparent prevalence. CRS: composite reference standard; FECT: formalin-ethyl acetate concentration technique.

The true prevalence in the study population by informed BLCM was 55.03% (95% CrIs: 50.06–60.08%) for *A. lumbricoides*, 66.45% (95% CrIs: 61.59–71.24%) for hookworm and 11.58% (95% CrIs: 9.04–14.51%) for *T. trichiura*. The use of vague priors had little impact on the results with CrIs largely overlapping (Figure 2; Online Appendix 2). Apparent prevalences based on a single microscopic test or CRS ranged from 27.35–48.61% for *A. lumbricoides*, from 21.43–67.94% for hookworm and from 0.07–13.41% for *T. trichiura* (Figure 2; Online Appendix 2). Combined with the intensity of infection data (Table 2), the study population can be described as having a high prevalence and low intensity of STH infections. Given the purposively sampled population these prevalence results should not be inferred beyond the six study villages to a wider population or region. Hierarchical BLCMs were considered to estimate village-specific prevalences; however, this approach resulted in sparse data in some villages that would have been challenging to manage analytically.

In terms of agreement between test results, the highest agreement was seen for *A. lumbricoides* with good agreement between McMaster2 and all other tests, and McMaster and Malachite; moderate agreement between FECT and Malachite, and FECT and McMaster. Agreement was poorer for hookworm and *T. trichiura* with poor agreement between Malachite and all other tests, fair agreement between FECT and McMaster, and FECT and McMaster2, and good agreement between McMaster and McMaster2 methods (Table 3).

**Table 3.** Pairwise diagnostic agreement by Kappa statistic

Parasite	Method	FECT	Malachite	McMaster	McMaster2
*A. lumbricoides*	FECT	1.00	–	–	–
	Malachite	0.55 (0.47–0.63)	1.00	–	–
	McMaster	0.59 (0.51–0.67)	0.62 (0.54–0.70)	1.00	–
	McMaster2	0.73 (0.65–0.81)	0.66 (0.58–0.74)	0.80 (0.72–0.88)	1.00
Hookworm	FECT	1.00	–	–	–
	Malachite	0.16 (0.09–0.23)	1.00	–	–
	McMaster	0.22 (0.14–0.30)	0.16 (0.08–0.24)	1.00	–
	McMaster2	0.23 (0.17–0.30)	0.19 (0.12–0.25)	0.79 (0.71–0.87)	1.00
*T. trichiura*	FECT	1.00	–	–	–
	Malachite	0.09 (0.02–0.15)	1.00	–	–
	McMaster	0.23 (0.17–0.30)	0.09 (0.06–0.13)	1.00	–
	McMaster2	0.23 (0.17–0.29)	0.09 (0.06–0.13)	1.00^1^ (0.92–1.00)	1.00

FECT: formalin-ethyl acetate concentration technique.

Kappa interpretation: K<0.20 poor; 0.21–0.40 fair; 0.41–0.60 moderate; 0.61–0.80 good; 0.81–1.00 very good agreement; –, corresponding pairwise result presented in another cell.

^1^All *T. trichiura* results equal for McMaster and McMaster2.

Considering only the traditional McMaster and novel McMaster2 method, the McMaster2 method resulted in a significantly higher prevalence of *A. lumbricoides* and hookworm (p<0.001) (Figure 2). An additional 52 infections of *A. lumbricoides* were detected by examining the bottom layer of the McMaster slide, representing an increase of 32.10% of infections that were detected by examining only the top layer. Similarly, 60 hookworm infections were detected only on the bottom layer, a 28.30% increase in infections detected (Online Appendix 2). All *T. trichiura* eggs were observed on the top layer meaning that McMaster and McMaster2 results were the same in all individuals, as reflected in the identical intensity categories (Table 2), apparent prevalence estimates (Figure 2) and perfect agreement between tests (Table 3). With respect to intensity, there was a strong correlation between McMaster and McMaster2 EPGs with Kendall's tau values of 0.85 for *A. lumbricoides* and 0.81 for hookworm. The arithmetic mean EPG was significantly higher for McMaster2 for both *A. lumbricoides* and hookworm (p<0.001) (Table 4).

**Table 4.** Comparison of mean eggs per gram by McMaster and McMaster2 methods

	*A. lumbricoides*	Hookworm	*T. trichiura*
McMaster	409.49	48.17	15.33
McMaster2	547.39*	74.83*	15.33^NS^

* Wilcoxon signed rank test (p<0.001).

^NS^All *T. trichiura* results were equal for McMaster and McMaster2 methods and there was no significant difference.

### Diagnostic sensitivity

**Figure 3.**
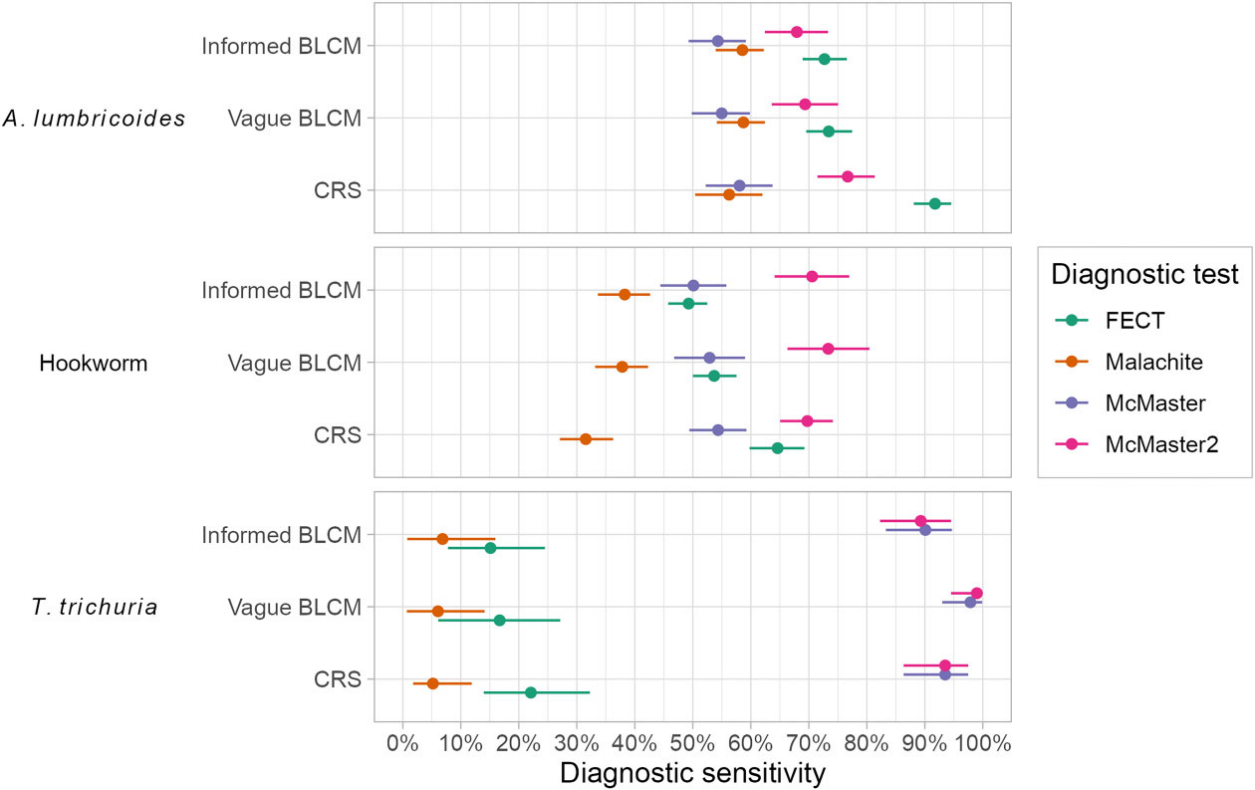
Sensitivity of formalin-ethyl acetate concentration technique (FECT), Malachite smear, McMaster and McMaster2 methods for the detection of soil-transmitted helminths using informed and vague Bayesian latent class models (BLCMs) and a composite reference standard (CRS). Dots represent medians for BLCMs and the mean for CRS. Lines represent 95% CrIs for BLCMs and the 95% CI for CRS.

The FECT and McMaster2 method were the best performing tests for *A. lumbricoides* with sensitivities of 72.70% (95% CrIs: 68.92–76.56%) and 67.93% (95% CrIs: 62.41–73.31%) estimated by informed BLCM. Sensitivity estimates for the Malachite smear and McMaster method were significantly lower at 58.54% (95% CrIs: 53.95–62.24%) and 54.32% (95% CrIs: 49.28–59.14%). The use of vague priors did not substantially alter these result with CrIs largely overlapping (Figure 3; Online Appendix 2). Using the CRS method provided substantially greater sensitivity estimates of 91.76% (95% CI 88.10–94.56%) and 76.70% (95% CI 71.48–81.37%) for the FECT and McMaster2 method. Meanwhile, CRS estimates for the Malachite smear (56.27%; 95% CI 50.41–62.00%) and McMaster method (58.06%; 95% CI 52.22–63.75%) were similar to the BLCM results (Figure 3; Online Appendix 2).

For hookworm, the McMaster2 method was the most sensitive test, with an estimate of 70.56% (95% CrIs: 64.10–76.96%) by informed BLCM. This was significantly greater than the FECT (49.28%, 95% CrIs: 45.76–52.49%) and McMaster method (50.11%, 95% CrIs: 44.40–55.78%). The Malachite smear was outperformed significantly by all other tests with an estimated sensitivity of 38.26% (95% CrIs: 33.63–42.63%). The use of vague priors did not markedly influence the results with all CrIs overlapping between vague and informed BLCMs (Figure 3; Online Appendix 2). The CRS method provided a similar estimate for the McMaster2 method (69.74%; 95% CI 65.05–74.14%), a slightly higher estimate for the McMaster method (54.36%; 95% CI 49.40–59.25%), a higher estimate for the FECT (64.62%; 95% CI 59.78–69.24%) and a lower estimate for the Malachite smear (31.54%; 95% CI 27.08–36.27%) (Figure 3; Online Appendix 2).

The McMaster and McMaster2 methods were overwhelmingly the most sensitive tests for *T. trichiura*, with sensitivities of 90.10% (95% CrIs: 83.29–94.67%) and 89.3% (95% CrIs: 82.28–94.52%) compared with 15.12% (95% CrIs: 7.79–24.50%) for FECT and 6.85% (95% CrIs: 0.77–15.96%) for the Malachite smear by informed BLCM (Figure 3; Online Appendix 2). Results and inference were relatively similar with the use of a vague BLCM or CRS (Figure 3; Online Appendix 2). This performance gap was not unexpected as the contingency tables informing the analyses included only one sample that was positive by all four tests, 57 samples that were McMaster and McMaster2 positive but FECT and Malachite smear negative, and only five samples that were McMaster and McMaster2 negative but positive by another test (Online Appendix 2).

## Discussion

The FECT and other formalin-fixed methods are regularly applied to large-scale STH surveys where there are insufficient resources to process fresh samples in an appropriate timeframe. There are other methods, such as the McMaster, that can also be applied to formalin-fixed samples. This study has described the McMaster2 method and found that it was the most sensitive diagnostic test for formalin-fixed STHs compared with the FECT, McMaster and Malachite smear. This suggests that the McMaster2 method on formalin-fixed samples may be a viable alternative to FECT and, like the McMaster, provides the added benefit of estimating the intensity of infection. Intensity of infection is linked to severity of disease and capturing EPG allows for mapping of intensity that may provide control programmes with more detailed information than prevalence alone.^28,29^ It is accepted that formalin-fixed methods are generally inferior to fresh analysis; however, these methods will continue to be required in many STH-endemic countries until there are substantial changes to diagnostic techniques, laboratory capacity and public health systems more broadly. The McMaster method has regularly been found to be faster to prepare and read compared with other methods. Even if the reading time for the McMaster2 is double that of the McMaster, due to reading both the top and bottom focal layers, it would still likely be faster or at least equivalent to other methods.^6,13,30^ This suggests it should be cost comparative; however, further research comparing the costs of different methods is required. The poor performance of the Malachite smear across all analyses means that it should not be considered useful for large-scale STH surveys and caution is suggested for the use of direct smears with formalin-fixed samples given their methodological similarities.

Although the Kato-Katz method is widely regarded as the most cost-effective and well-known faecal microscopy technique for STHs, it is not always feasible in all settings. Field conditions should be assessed prior to choosing a diagnostic test and it is expected that there are many field surveys conducted using the Kato-Katz method that are analysed outside of the recommended guidelines.^8^ This is particularly relevant for hookworm, where worms rapidly hatch from eggs in a matter of hours, unless fixed, and the glycerol used in the Kato-Katz method may damage hookworm eggs over time.^31,32^ Formalin-fixation reduces this time pressure; however, one study into the effect of formalin-fixation on the mini-FLOTAC method demonstrated that hookworm eggs become deformed after approximately 2 wk in storage.^33^ Another diagnostic study examined the sensitivity of Kato-Katz, McMaster and FLOTAC methods with both fresh samples and samples fixed in formalin for 6 mo.^11^ It found no difference in sensitivity between fresh and fixed samples for *A. lumbricoides* and *T. trichiura*, while for hookworm the median sensitivity of FLOTAC and McMaster methods on formalin-fixed samples was approximately 10 and 15 percentage points less than the fresh method; however, 95% CIs did overlap in both cases, indicating a lack of statistical significance. Our study extended this formalin-fixed storage period substantially without seeing a substantial decrease in sensitivity estimates.

Previous global meta-analysis of faecal microscopy performance applied similar methods to this study and reported fresh McMaster method sensitivities in low-intensity settings of 48.90% (95% CrIs: 37.20–58.90%) for *A. lumbricoides*, 34.50% (95% CrIs: 27.90–42.0%) for hookworm and 75.50% (95% CrIs: 70.0–80.40%) for *T. trichiura*. The sensitivity of our formalin-fixed McMaster method was similar for *A. lumbricoides* (54.32%, 95% CrIs: 49.28–59.14%) and greater for hookworm (50.11%, 95% CrIs: 44.40–55.78%) and *T. trichiura* (90.10%, 95% CrIs: 83.29–94.67%), suggesting that the impact of formalin-fixed may not be as great as anecdotally believed. It must be stressed that this is a purely exploratory discussion as there may be other factors influencing this comparison beyond fixation and an update of past global meta-analysis is encouraged, including consideration of sample fixation, along with further prospective studies that examine fresh and fixed methods in parallel, such as Albonico, Rinaldi^11^ and Barda, Albonico.^33^

The McMaster method has been modified many times over history. Modifications have included the use of different flotation solutions, the number of chambers counted, the area examined and the time allowed for flotation, among others.^34–37^ The anecdotal experience of the authors is that the longer a McMaster slide is rested before reading to allow for flotation of eggs, the more sensitive it becomes. However, extended flotation times lead to longer staff hours and increased cost during large surveys and risk damaging fragile parasite eggs. The McMaster2 method described in this paper takes an alternative approach to increasing the sensitivity of the McMaster method by examining both the top and bottom focal layers. This increases the reading time rather than the flotation time and was more sensitive than the traditional McMaster method. As long as the McMaster slide is kept clean and the faecal solution is contained only within the McMaster chamber, the multiplication factor to estimate intensity of infection for the McMaster2 method should remain the same as for the McMaster method. The same volume of solution is examined; simply, a more complete examination is made.

Intensity of infection plays an important role in diagnostic sensitivity and has been suggested as a reason for differing performance across populations and studies.^11^ In this study, most individuals were infected at low intensity and the impact of intensity on test sensitivity could not be assessed. The number of moderate and high-intensity infections were low enough that their removal did not substantially impact the results in informal analysis and their inclusion makes this study an analysis of a practical low-intensity setting. The FECT, Malachite smear, McMaster and McMaster2 methods were expected to perform more equally across all STHs, as was the case with the FECT, fresh direct smear and fresh McMaster method in global meta-analysis.^10^ The differences observed in this study may be explained by the nature of the tests and low intensity of infections. In terms of diagnostic tests, the FECT relies on sedimentation, Malachite smear on direct observation and the McMaster and McMaster2 methods on flotation.^8,15^ First principles support our finding of poor Malachite smear performance across all STHs as it directly examines only a small amount of faeces compared with the concentration methods and this performance gap will be exacerbated in low-intensity settings. The sedimentary nature of the FECT and examining the bottom layer during the McMaster2 method means that these tests may be expected to have superior performance for heavier eggs, such as infertile *A. lumbricoides*. This was supported by our results, where the FECT and McMaster2 method displayed significantly higher sensitivity for *A. lumbricoides* compared with the Malachite smear and McMaster method. Infertile and fertile *A. lumbricoides* were not recorded separately in this study; however, the FECT technicians informally reported regularly observing infertile *A. lumbricoides*, while the McMaster, McMaster2 and Malachite technicians recalled relatively few. When infertile *A. lumbricoides* were detected by McMaster2, they were often believed to be on the bottom layer of the slide, meaning that they would have been missed by the traditional McMaster.

The difference in sensitivity between tests for *T. trichiura* was marked in this study. However, the results are relatively consistent with the global meta-analysis restricted to low-intensity *T. trichiura* settings.^10^ The sensitivity was significantly lower for fresh direct smear and FECT compared with fresh McMaster, with estimates of 14.90% (95% CrIs: 0.50–48.60%) and 21.50% (95% CrIs: 10.60–32.90%) compared with 75.50% (95% CrIs: 70.00–80.40%).^10^ The difference in performance between the FECT and McMaster and McMaster2 methods for *T. trichiura* in our study and the global meta-analysis may be described by several factors. First, there may potentially be a loss of eggs during the FECT sedimentation process, as is believed to be the case for *Schistosoma*.^38^ This loss may only be noticeable in low-intensity settings and such a marked difference was not seen in the global meta-analysis when data were not stratified by intensity. Similarly, low-intensity *T. trichiura* infections have fewer EPG and there is a smaller probability of agreement as to whether an egg is transferred to a sample pot or slide for examination. The difference in volume or mass of faeces examined may also create a disparity between the methods at low intensities. The FECT processes approximately 1 g of faeces and examines one drop of sediment that is not weighed. The McMaster and McMaster2 methods process a weighed 2 g of faeces and examine the equivalent of homogenised 0.02 g. Alternatively, floating *T. trichiura* eggs may simply be more effective than sinking them.

Other factors may have influenced this study's findings; however, these are more generic and should have affected all results to a similar degree. For example, there may be increased debris during the FECT, Malachite smear and McMaster2 method compared with the McMaster method. This may make egg detection more challenging and decrease the sensitivity of these methods. Anecdotally, the laboratory technicians regularly reported that the bottom layer of the McMaster2 method was difficult to read due to debris. Including straining in the preparation of the McMaster2 method may further improve its performance. Given that two laboratories and several technicians conducted the different tests, there is the possibility that technical skill rather than inherent difference between the tests is represented in these results. This is not expected to have had a substantial impact as the decision to use two laboratories was made because each was well equipped and experienced in performing their respective tests. Finally, it must be remembered that diagnostic test performance is specific to a particular context and it is a fundamental axiom that test performance will vary depending on factors such as the population, laboratory and local epidemiological conditions.

This study applied two different approaches to evaluating diagnostic test performance when there is no gold standard. The BLCM approach is strongly recommended and the informed BLCM results should be considered the most reliable from this study. The exaggerated estimates using the CRS method in this study demonstrates its fallacies. The CRS estimate for the sensitivity of the FECT for *A. lumbricoides* was overestimated (91.76%; 95% CI 88.10–94.56%) and implausible when considering the general performance of faecal microscopy for the detection of STHs. The CRS method has been commonly used when no gold standard is available; however, the method is fraught with issues of interpretation, particularly across studies and when all tests perform relatively poorly, as is the case with faecal microscopy.^23^ Informed BLCMs incorporate prior knowledge on test performance and prevalence, and balance this with new empirical data. Vague BLCMs often produce results more analogous to frequentist methods with the data speaking for itself; however, the aspect of building on what is already known and incorporating prior knowledge is lost. In this study, there was very little difference between informed and vague BLCMs due to the broad prior distributions that were used in the informed BLCMs. The use and reporting of BLCMs is now standardised and widely accepted for evaluating the performance of diagnostic tests.^21,39^

## Conclusions

When formalin-fixation is required for large-scale STH surveys, the McMaster2 method should be considered as a viable alternative to the routinely performed FECT. It requires similar investment and resources to other formalin-fixed methods and is advantageous over the FECT in that it can provide intensity of infection and does not require the use of hazardous reagents in the laboratory. Re-examining formalin-fixed methods and the influence of formalin-fixation on faecal microscopy methods may be a worthwhile endeavour to improve practical guidelines for endemic country use while more sophisticated diagnostic tools are still in development.

## Supplementary Material

traf011_Supplemental_Files

## Data Availability

The dataset supporting the conclusions of this article is included within the article (and its supplementary data) or is available from the authors upon reasonable request.

## References

[1] Bethony J, Brooker S, Albonico M et al. Soil-transmitted helminth infections: ascariasis, trichuriasis, and hookworm. Lancet. 2006;367(9521):1521–32. 16679166 10.1016/S0140-6736(06)68653-4

[2] Turner HC, Stolk WA, Solomon AW et al. Are current preventive chemotherapy strategies for controlling and eliminating neglected tropical diseases cost-effective? BMJ Glob Health. 2021;6(8):e005456. 10.1136/bmjgh-2021-005456 PMC8362715 34385158

[3] WHO . Global Report on Neglected Tropical Diseases 2023. Geneva, Switzerland: WHO, 2023.

[4] Institute for Health Metrics and Evaluation . 2024. GBD Results. Available at: https://vizhub.healthdata.org/gbd-results/ [accessed 2024].

[5] WHO . Guideline: Preventive Chemotherapy to Control Soil-Transmitted Helminth Infections in at-Risk Population Groups. Geneva, Switzerland: WHO, 2017. 29578660

[6] Speich B, Knopp S, Mohammed KA et al. Comparative cost assessment of the Kato-Katz and FLOTAC techniques for soil-transmitted helminth diagnosis in epidemiological surveys. Parasit Vectors. 2010;3(1):71. 20707931 10.1186/1756-3305-3-71 PMC2936391

[7] WHO . Bench Aids for the Diagnosis of Intestinal Parasites. Geneva, Switzerland: World Health Organization, 1994.

[8] WHO . Bench Aids for the Diagnosis of Intestinal Parasites. Geneva, Switzerland: World Health Organization, 2019.

[9] Phonekeo S, Kounnavong S, Vonglokham M et al. Intestinal helminth infections and associated risk factors among adults in the Lao People's Democratic Republic. Infect Dis Poverty. 2023;12(1): 10.1186/s40249-023-01112-0 PMC10311807 37386528

[10] Nikolay B, Brooker SJ, Pullan RL. Sensitivity of diagnostic tests for human soil-transmitted helminth infections: a meta-analysis in the absence of a true gold standard. Int J Parasitol. 2014;44(11):765–74. 24992655 10.1016/j.ijpara.2014.05.009 PMC4186778

[11] Albonico M, Rinaldi L, Sciascia S et al. Comparison of three copromicroscopic methods to assess albendazole efficacy against soil-transmitted helminth infections in school-aged children on Pemba Island. Trans R Soc Trop Med Hyg. 2013;107(8):493–501. 23843559 10.1093/trstmh/trt051

[12] Adugna S, Kebede T, Mekonnen Z et al. Diagnostic performance of Mini Parasep® solvent-free faecal parasite concentrator relative to Kato-Katz and McMaster for the diagnosis of intestinal parasitic infections. Trans R Soc Trop Med Hyg. 2017;111(12):572–8. 29509952 10.1093/trstmh/try010 PMC6543883

[13] Barda B, Cajal P, Villagran E et al. Mini-FLOTAC, Kato-Katz and McMaster: three methods, one goal; highlights from north Argentina. Parasit Vectors. 2014;7(1):271. 24929554 10.1186/1756-3305-7-271 PMC4074144

[14] Levecke B, Behnke JM, Ajjampur SSR et al. A comparison of the sensitivity and fecal egg counts of the McMaster egg counting and Kato-Katz thick smear methods for soil-transmitted helminths. PLoS Negl Trop Dis. 2011;5(6):e1201. 21695104 10.1371/journal.pntd.0001201 PMC3114752

[15] Elliot A, Morgan UM, Thompson RCA. Improved staining method for detecting cryptosporidium oocysts in stools using malachite green. J Gen Appl Microbiol. 1999;45(3):139–42. 12501386 10.2323/jgam.45.139

[16] Larkins A, Knight B, Keokhamphavanh B et al. Sensitivity and specificity of microscopic and molecular techniques for the diagnosis of taeniasis. Acta Trop. 2024;260:107414. 39362512 10.1016/j.actatropica.2024.107414

[17] Brown LD, Cai TT, Dasgupta A. Interval estimation for a binomial proportion. Stat Sci. 2001;16(2):101–33.

[18] Stevenson M, Nunes T, Heuer C et al. epiR: an R Package for the Analysis of Epidemiological Data. 2024, https://cran.r-project.org/web/packages/epiR.

[19] WHO . Diagnostic Target Product Profile for Monitoring and Evaluation of Soil-transmitted Helminth Control Programmes. Geneva, Switzerland: World Health Organization, 2021.

[20] Altman DG, Bland JM. Diagnostic tests 1: sensitivity and specificity. BMJ. 1994;308(6943):1552. 8019315 10.1136/bmj.308.6943.1552 PMC2540489

[21] Umemneku Chikere CM, Wilson K, Graziadio S et al. Diagnostic test evaluation methodology: a systematic review of methods employed to evaluate diagnostic tests in the absence of gold standard—An update. PLoS One. 2019;14(10):e0223832. 31603953 10.1371/journal.pone.0223832 PMC6788703

[22] Joseph L, Gyorkos TW, Coupal L. Bayesian estimation of disease prevalence and the parameters of diagnostic tests in the absence of a gold standard. Am J Epidemiol. 1995;141(3):263–72. 7840100 10.1093/oxfordjournals.aje.a117428

[23] Dendukuri N, Joseph L. Bayesian approaches to modeling the conditional dependence between multiple diagnostic tests. Biometrics. 2001;57(1):158–67. 11252592 10.1111/j.0006-341x.2001.00158.x

[24] Menten J, Boelaert M, Lesaffre E. Bayesian latent class models with conditionally dependent diagnostic tests: a case study. Stat Med. 2008;27(22):4469–88. 18551515 10.1002/sim.3317

[25] Speich B, Ali SM, Ame SM et al. Quality control in the diagnosis of trichuris trichiura and ascaris lumbricoides using the Kato-Katz technique: experience from three randomised controlled trials. Parasit Vectors. 2015;8(1):82. 25652120 10.1186/s13071-015-0702-z PMC4326492

[26] Lunn D, Best N, Jackson C et al. The Bugs Book: a Practical Introduction to Bayesian Analysis Boca Raton, FL: Chapman and Hall/CRC, an imprint of Taylor and Francis, 2012.

[27] RStudio Team . RStudio: Integrated Development for R. Boston, MA: RStudio, PBC, 2021.

[28] Forrer A, Vounatsou P, Sayasone S et al. Risk profiling of hookworm infection and intensity in Southern Lao People's Democratic Republic using bayesian models. PLoS Negl Trop Dis. 2015;9(3): 10.1371/journal.pntd.0003486 PMC4378892 25822794

[29] Campbell SJ, Nery SV, Doi SA et al. Complexities and perplexities: a critical appraisal of the evidence for soil-transmitted helminth infection-related morbidity. PLoS Negl Trop Dis. 2016;10(5):e0004566. 27196100 10.1371/journal.pntd.0004566 PMC4873196

[30] Levecke B, De Wilde N, Vandenhoute E et al. Field validity and feasibility of four techniques for the detection of trichuris in simians: a model for monitoring drug efficacy in public health? PLoS Negl Trop Dis. 2009;3(1):e366. 19172171 10.1371/journal.pntd.0000366 PMC2621347

[31] Bosch F, Palmeirim MS, Ali SM et al. Diagnosis of soil-transmitted helminths using the Kato-Katz technique: what is the influence of stirring, storage time and storage temperature on stool sample egg counts? PLoS Negl Trop Dis. 2021;15(1):e0009032. 33481808 10.1371/journal.pntd.0009032 PMC7857572

[32] Dacombe RJ, Crampin AC, Floyd S et al. Time delays between patient and laboratory selectively affect accuracy of helminth diagnosis. Trans R Soc Trop Med Hyg. 2007;101(2):140–5. 16824566 10.1016/j.trstmh.2006.04.008

[33] Barda B, Albonico M, Ianniello D et al. How long can stool samples Be fixed for an accurate diagnosis of soil-transmitted helminth infection using mini-FLOTAC? PLoS Negl Trop Dis. 2015;9(4):e0003698–e. 25848772 10.1371/journal.pntd.0003698 PMC4388498

[34] Cringoli G, Rinaldi L, Veneziano V et al. The influence of flotation solution, sample dilution and the choice of McMaster slide area (volume) on the reliability of the McMaster technique in estimating the faecal egg counts of gastrointestinal strongyles and dicrocoelium dendriticum in sheep. Vet Parasitol. 2004;123(1):121–31. 15265576 10.1016/j.vetpar.2004.05.021

[35] Dunn A, Keymer A. Factors affecting the reliability of the McMaster technique. J Helminthol. 1986;60(4):260–2. 3794288 10.1017/s0022149x00008464

[36] Pereckienė A, Kaziūnaitė V, Vyšniauskas A et al. A comparison of modifications of the McMaster method for the enumeration of Ascaris suum eggs in pig faecal samples. Vet Parasitol. 2007;149(1):111–6. 17703889 10.1016/j.vetpar.2007.04.014

[37] Vadlejch J, Petrtýl M, Zaichenko I et al. Which McMaster egg counting technique is the most reliable? Parasitol Res. 2011;109(5):1387–94. 21526406 10.1007/s00436-011-2385-5

[38] Lier T, Simonsen GS, Wang T et al. Low sensitivity of the formol-ethyl acetate sedimentation concentration technique in Low-intensity schistosoma japonicum infections. PLoS Negl Trop Dis. 2009;3(2):e386. 19238192 10.1371/journal.pntd.0000386 PMC2638014

[39] Kostoulas P, Nielsen SS, Branscum AJ et al. Reporting guidelines for diagnostic accuracy studies that use bayesian latent class models (STARD-BLCM). Stat Med. 2017;36(23):3603–4. 28675923 10.1002/sim.7316

