# Supplementary material for: Diagnostic sensitivity of formalin-fixed faecal microscopy for the detection of soil-transmitted helminths
Source: Trans R Soc Trop Med Hyg. 2025 Feb 8;119(6):596–605. doi: 10.1093/trstmh/traf011 (PMC12138886; doi:10.1093/trstmh/traf011)
Supplement: traf011_Supplemental_Files [file traf011_supplemental_files.zip › Appendix 1. BLCM description.docx]

# Appendix 2. Bayesian latent class model description

#=== LIKELIHOOD ===#

n[1:16] ~ dmulti(p[1:16], 574)

p[1] <- Prev*((1-Se_FECT)*(1-Se_Malachite)*(1-Se_McMaster)*(1-Se_McMaster2)+covpAB+covpAC+covpAD+covpBC+covpBD+covpCD) +

(1-Prev)*(Sp_fixed*Sp_fixed*Sp_fixed*Sp_fixed+covnAB+covnAC+covnAD+covnBC+covnBD+covnCD) #----

p[2] <- Prev*((1-Se_FECT)*(1-Se_Malachite)*(1-Se_McMaster)*Se_McMaster2+covpAB+covpAC-covpAD+covpBC-covpBD-covpCD) +

(1-Prev)*(Sp_fixed*Sp_fixed*Sp_fixed*(1-Sp_fixed)+covnAB+covnAC-covnAD+covnBC-covnBD-covnCD) #---+

p[3] <- Prev*((1-Se_FECT)*(1-Se_Malachite)*Se_McMaster*(1-Se_McMaster2)+covpAB-covpAC+covpAD-covpBC+covpBD-covpCD) +

(1-Prev)*(Sp_fixed*Sp_fixed*(1-Sp_fixed)*Sp_fixed+covnAB-covnAC+covnAD-covnBC+covnBD-covnCD) #--+-

p[4] <- Prev*((1-Se_FECT)*(1-Se_Malachite)*Se_McMaster*Se_McMaster2+covpAB-covpAC-covpAD-covpBC-covpBD+covpCD) +

(1-Prev)*(Sp_fixed*Sp_fixed*(1-Sp_fixed)*(1-Sp_fixed)+covnAB-covnAC-covnAD-covnBC-covnBD+covnCD) #--++

p[5] <- Prev*((1-Se_FECT)*Se_Malachite*(1-Se_McMaster)*(1-Se_McMaster2)-covpAB+covpAC+covpAD-covpBC-covpBD+covpCD) +

(1-Prev)*(Sp_fixed*(1-Sp_fixed)*Sp_fixed*Sp_fixed-covnAB+covnAC+covnAD-covnBC-covnBD+covnCD) #-+--

p[6] <- Prev*((1-Se_FECT)*Se_Malachite*(1-Se_McMaster)*Se_McMaster2-covpAB+covpAC-covpAD-covpBC+covpBD-covpCD) +

(1-Prev)*(Sp_fixed*(1-Sp_fixed)*Sp_fixed*(1-Sp_fixed)-covnAB+covnAC-covnAD-covnBC+covnBD-covnCD) #-+-+

p[7] <- Prev*((1-Se_FECT)*Se_Malachite*Se_McMaster*(1-Se_McMaster2)-covpAB-covpAC+covpAD+covpBC-covpBD-covpCD) +

(1-Prev)*(Sp_fixed*(1-Sp_fixed)*(1-Sp_fixed)*Sp_fixed-covnAB-covnAC+covnAD+covnBC-covnBD-covnCD) #-++-

p[8] <- Prev*((1-Se_FECT)*Se_Malachite*Se_McMaster*Se_McMaster2-covpAB-covpAC-covpAD+covpBC+covpBD+covpCD) +

(1-Prev)*(Sp_fixed*(1-Sp_fixed)*(1-Sp_fixed)*(1-Sp_fixed)-covnAB-covnAC-covnAD+covnBC+covnBD+covnCD) #-+++

p[9] <- Prev*(Se_FECT*(1-Se_Malachite)*(1-Se_McMaster)*(1-Se_McMaster2)-covpAB-covpAC-covpAD+covpBC+covpBD+covpCD) +

(1-Prev)*((1-Sp_fixed)*Sp_fixed*Sp_fixed*Sp_fixed-covnAB-covnAC-covnAD+covnBC+covnBD+covnCD) #+---

p[10] <-Prev*(Se_FECT*(1-Se_Malachite)*(1-Se_McMaster)*Se_McMaster2-covpAB-covpAC+covpAD+covpBC-covpBD-covpCD) +

(1-Prev)*((1-Sp_fixed)*Sp_fixed*Sp_fixed*(1-Sp_fixed)-covnAB-covnAC+covnAD+covnBC-covnBD-covnCD) #+--+

p[11] <- Prev*(Se_FECT*(1-Se_Malachite)*Se_McMaster*(1-Se_McMaster2)-covpAB+covpAC-covpAD-covpBC+covpBD-covpCD) +

(1-Prev)*((1-Sp_fixed)*Sp_fixed*(1-Sp_fixed)*Sp_fixed-covnAB+covnAC-covnAD-covnBC+covnBD-covnCD) #+-+-

p[12] <- Prev*(Se_FECT*(1-Se_Malachite)*Se_McMaster*Se_McMaster2-covpAB+covpAC+covpAD-covpBC-covpBD+covpCD) +

(1-Prev)*((1-Sp_fixed)*Sp_fixed*(1-Sp_fixed)*(1-Sp_fixed)-covnAB+covnAC+covnAD-covnBC-covnBD+covnCD) #+-++

p[13] <- Prev*(Se_FECT*Se_Malachite*(1-Se_McMaster)*(1-Se_McMaster2)+covpAB-covpAC-covpAD-covpBC-covpBD+covpCD) +

(1-Prev)*((1-Sp_fixed)*(1-Sp_fixed)*Sp_fixed*Sp_fixed+covnAB-covnAC-covnAD-covnBC-covnBD+covnCD) #++--

p[14] <- Prev*(Se_FECT*Se_Malachite*(1-Se_McMaster)*Se_McMaster2+covpAB-covpAC+covpAD-covpBC+covpBD-covpCD) +

(1-Prev)*((1-Sp_fixed)*(1-Sp_fixed)*Sp_fixed*(1-Sp_fixed)+covnAB-covnAC+covnAD-covnBC+covnBD-covnCD) #++-+

p[15] <- Prev*(Se_FECT*Se_Malachite*Se_McMaster*(1-Se_McMaster2)+covpAB-covpAC-covpAD-covpBC-covpBD-covpCD) +

(1-Prev)*((1-Sp_fixed)*(1-Sp_fixed)*(1-Sp_fixed)*Sp_fixed+covnAB+covnAC-covnAD+covnBC-covnBD-covnCD) #+++-

p[16] <- Prev*(Se_FECT*Se_Malachite*Se_McMaster*Se_McMaster2+covpAB+covpAC+covpAD+covpBC+covpBD+covpCD) +

(1-Prev)*((1-Sp_fixed)*(1-Sp_fixed)*(1-Sp_fixed)*(1-Sp_fixed)+covnAB+covnAC+covnAD+covnBC+covnBD+covnCD) #++++

#=== PRIOR ===#

Prev ~ dbeta(prev.shape1, prev.shape2) ## Prior for Prev

Se_FECT ~ dbeta(FECT.shape1, FECT.shape2) ## Prior for Se of Test A

Se_Malachite ~ dbeta(Malachite.shape1, Malachite.shape2) ## Prior for Se of Test B

Se_McMaster ~ dbeta(McMaster.shape1, McMaster.shape2) ## Prior for Se of Test C

Se_McMaster2 ~ dbeta(McMaster2.shape1, McMaster2.shape2) ## Prior for Se of Test D

#=== CONDITIONAL DEPENDENCE STRUCTURE ===#

covpAB ~ dunif(minpAB,maxpAB)

covpAC ~ dunif(minpAC,maxpAC)

covpAD ~ dunif(minpAD,maxpAD)

covpBC ~ dunif(minpBC,maxpBC)

covpBD ~ dunif(minpBD,maxpBD)

covpCD ~ dunif(minpCD,maxpCD)

covnAB ~ dunif(minnAB,maxnAB)

covnAC ~ dunif(minnAC,maxnAC)

covnAD ~ dunif(minnAD,maxnAD)

covnBC ~ dunif(minnBC,maxnBC)

covnBD ~ dunif(minnBD,maxnBD)

covnCD ~ dunif(minnCD,maxnCD)

minpAB <- (1-Se_FECT)*(Se_Malachite-1)

maxpAB <- min(Se_FECT,Se_Malachite) - Se_FECT*Se_Malachite

minpAC <- (1-Se_FECT)*(Se_McMaster-1)

maxpAC <- min(Se_FECT,Se_McMaster) - Se_FECT*Se_McMaster

minpAD <- (1-Se_FECT)*(Se_McMaster2-1)

maxpAD <- min(Se_FECT,Se_McMaster2) - Se_FECT*Se_McMaster2

minpBC <- (1-Se_Malachite)*(Se_McMaster-1)

maxpBC <- min(Se_Malachite,Se_McMaster) - Se_Malachite*Se_McMaster

minpBD <- (1-Se_Malachite)*(Se_McMaster2-1)

maxpBD <- min(Se_Malachite,Se_McMaster2) - Se_Malachite*Se_McMaster2

minpCD <- (1-Se_McMaster)*(Se_McMaster2-1)

maxpCD <- min(Se_McMaster,Se_McMaster2) - Se_McMaster*Se_McMaster2

minnAB <- (Sp_fixed-1)*(1-Sp_fixed)

maxnAB <- Sp_fixed - Sp_fixed*Sp_fixed

minnAC <- (Sp_fixed-1)*(1-Sp_fixed)

maxnAC <- Sp_fixed - Sp_fixed*Sp_fixed

minnAD <- (Sp_fixed-1)*(1-Sp_fixed)

maxnAD <- Sp_fixed - Sp_fixed*Sp_fixed

minnBC <- (Sp_fixed-1)*(1-Sp_fixed)

maxnBC <- Sp_fixed - Sp_fixed*Sp_fixed

minnBD <- (Sp_fixed-1)*(1-Sp_fixed)

maxnBD <- Sp_fixed - Sp_fixed*Sp_fixed

minnCD <- (Sp_fixed-1)*(1-Sp_fixed)

maxnCD <- Sp_fixed - Sp_fixed*Sp_fixed
